# Review of the genus *Hierodula* Burmeister (Mantodea: Mantidae) in Iran

**DOI:** 10.3389/finsc.2026.1757219

**Published:** 2026-02-03

**Authors:** Zohreh Mirzaee, Martin Wiemers, Thomas Schmitt, Valeriy Govorov, Evgeny Shcherbakov

**Affiliations:** 1 Senckenberg Deutsches Entomologisches Institut, Müncheberg, Germany; 2 Entomology and Biogeography, Institute of Biochemistry and Biology, Faculty of Science, University of Potsdam, Potsdam, Germany; 3 Department of Zoology, Faculty of Science, Charles University, Prague, Czechia; 4 Department of Entomology, Faculty of Biology, Lomonosov Moscow State University, Moscow, Russia

**Keywords:** biogeography, integrative taxonomy, Iran, mantid, systematics

## Abstract

**Introduction:**

*Hierodula* is a morphologically conservative mantid genus with a complex taxonomic history and several problematic species-level concepts across its native and invaded ranges. In Iran, four nominal *Hierodula* species have historically been reported (*H. macrostigmata, H. tenuidentata, H. transcaucasica*, and “*H. trimacula*”), but their validity and distributions have remained uncertain due to overlapping diagnostic characters and limited molecular data. This study addresses these issues by reassessing all available Iranian material within an integrative framework.

**Methods:**

The revision combines: Morphological examination of type and non-type material from Iran, India, Pakistan, and Oman, including detailed study of external characters and male genitalia. Mitochondrial COI barcoding of *Hierodula* specimens from multiple Iranian provinces and Pakistan, analyzed with Bayesian inference, maximum likelihood, and model-based genetic distance estimation. Compilation and critical validation of distributional data from museum collections, literature, and iNaturalist records, followed by mapping in QGIS.

**Results:**

Morphological comparisons show that the holotype of *H. macrostigmata* and recently collected southern Iranian specimens are indistinguishable from *H. coarctata* in forewing stigma, pronotal shape, and male genitalia, supporting their synonymy. COI phylogenies and TN93 genetic distances recover two deeply divergent, well-supported clades corresponding to *H. coarctata* and the *H. tenuidentata* complex, with minimal intraspecific divergence and no separation between Iranian “*H. macrostigmata*” and Indian/Pakistani *H. coarctata*. Re-examination of specimens and literature demonstrates that records of “*Hierodula/Sphodromantis trimacula*” from Iran lack verifiable material, while male genital characters place the species unambiguously in Sphodromantis and confirm its absence from the Iranian fauna.

**Discussion:**

The integrative evidence indicates that only *H. coarctata* and *H. tenuidentata* are currently valid *Hierodula* species in Iran, with *H. macrostigmata* as a junior synonym of *H. coarctata* and previous Iranian reports of *S. trimacula* rejected. The clear molecular separation between *H. coarctata* and the *H. tenuidentata* complex, combined with broad morphological variability in traits such as forefemoral spine coloration, underscores the need to abandon historically overemphasized colour characters and highlights the utility of COI barcoding in resolving conservative mantid lineages. Remaining uncertainty regarding the status of *H. transcaucasica* versus *H. tenuidentata* at a broader Eurasian scale calls for a forthcoming multi-locus, range-wide revision to formally resolve their taxonomy.

## 1 Introduction

The insect order Mantodea, commonly known as praying mantises, represents a highly diverse group of predatory insects comprising more than 2,400 described species worldwide (1). Primarily distributed in tropical and subtropical regions, but also found in temperate zones, mantises play a pivotal role in regulating insect populations, thereby contributing significantly to the balance of various terrestrial ecosystems (2). Within Mantodea, members of the genus *Hierodula* Burmeister, 1838, are often referred to as giant Asian mantises. Species within this genus are notable for their large body size and vibrant coloration. Although the genus, as it is currently understood, is widespread across Australasia, recent taxonomic revisions and molecular studies have revealed a complex evolutionary history within it, suggesting that its traditional circumscription may encompass several distinct evolutionary lineages (3–7). This study combines morphological, molecular, and ecological evidence to clarify the taxonomy, distribution, and ecological roles of the *Hierodula* species in Iran. To date, four species have been recorded in Iran: *Hierodula macrostigmata* Deeleman-Reinhold, 1957 (8), *Hierodula tenuidentata* Saussure, 1869 (9), *Hierodula transcaucasica* Brunner von Wattenwyl, 1878 (10), and *Hierodula trimacula* Saussure, 1870 (11, 12). In this article, we review and assess the validity of these species in Iran, provide updated distribution maps, and discuss their status, synonymizing taxa where appropriate.

## 2 Materials and methods

### 2.1 Data collection

We utilized multiple types of data by combining occurrence records from published studies, museum specimens, recently collected field samples from various regions of Iran (all of which were examined or collected by the first author), and observation records from iNaturalist (https://www.inaturalist.org/observations?place_id=6818&taxon_id=328038). iNaturalist records of *Hierodula* were treated as complementary to the field and museum data in order to better cover the overall distribution of the genus across Iran. To retrieve these data, we searched the platform using the keyword “*Hierodula*” in combination with “Iran” and downloaded all matching observations with their associated photographs and metadata. All images were then inspected individually by the first author, and only those observations that clearly showed the diagnostic external characteristics of Iranian *Hierodula* species—most notably the presence and the form of the stigma on the forewing, together with the characteristic shape of the head and pronotum—were considered reliable and retained. Records lacking clear views of these structures or for which the characteristics could not be evaluated with sufficient confidence were excluded. All accepted observations were compiled in a spreadsheet provided as **Supplementary File 1**. We critically reviewed the morphological, molecular, and distribution evidence and complemented these with novel ecological and biological observations. These records were carefully checked for correct identification) (**Supplementary File 1**). Google Earth v. 9.174.0.2 (https://earth.google.com/web/) was used to georeference the specimens without coordinates based on the information present on the corresponding labels. A total of 129 records were obtained and plotted on a distribution map using QGIS v. 3.22. The complete list of records for each species is reported in **Supplementary File 1**.

### 2.2 Biology and life cycle

Live specimens and oothecae were collected over a 2-year period (2018–2020) from different districts in different provinces of Iran (details in **Supplementary File 1**). Sampling methods included sweep netting and hand-picking during the day through careful observation. Once collected, the specimens were put in separate plastic containers and later placed in separate plastic jars (15 cm × 15 cm × 10 cm) upon arrival at the laboratory. Some rocks and sticks were added to the containers in order to help the mantises climb and hang, especially during molting.

The specimens in the laboratory were observed throughout their entire life cycle (7 months, from March 2021 to September 2021). Oothecae were collected to record the following data: number of hatching nymphs, number of instars reaching the adult stage, and male-to-female ratio per ootheca. The individuals were kept at room temperature (25–27°C), with a relative air humidity (RH) of 50%–55% by misting the room on a regular basis. An HTC2 digital terrarium hygrometer (Dongguan, China) was used to monitor RH. Laboratory specimens were initially fed with one to two fruit flies (*Drosophila melanogaster* Meigen, 1830) every third day. Later instars were fed with living mealworm larvae (*Tenebrio molitor* Linnaeus, 1758) twice a week.

After completing their life cycles, the individuals were preserved in 96% ethanol for morphological and molecular studies. Specimens were examined, and measurements were taken, under a Leica M205 C stereomicroscope with an ocular micrometer. The classification system used in this study follows Schwarz and Roy (3). The descriptive terminology for the external morphology of the male and female, along with the preparation of the male genitalia, follows Brannoch et al. (13). The final segments of the male abdomen were dissected under a stereomicroscope in order to separate the genitalia from the terminalia. The genitalia were macerated as a whole in a 10% KOH solution. Subsequently, they were washed first with distilled water, then in ethanol (70%), and finally in glycerol for 24 h in order to ensure the complete removal of ethanol. Finally, the prepared genitalia were placed in a vial with glycerol drops for further study. The specimens were photographed with a system that included a Stone Master Stack Unit, an Olympus OM-D E-M1 Mark II camera, and Zeiss Luminar lenses (16, 25, 40, and 63 mm). Olympus Capture and Stone Master v.3.8 software were used to acquire the digital photos. Stacking was performed with Helicon Focus (v.7.6.1), and a scale bar was added with ImageJ (v.1.53t).

The material used in this study is preserved in the following collections: ESPC (personal collection, Evgeny Shcherbakov, Ramenskoye, Russia), SDEI (Senckenberg German Entomological Institute, Müncheberg, Germany), ZMPC (personal collection, Zohreh Mirzaee, Strausberg, Germany), and NMPC (National Museum, Prague, Czech Republic).

The abbreviations of the zoological institutes and museums mentioned in this study are as follows:

MHNG: Muséum d’Histoire naturelle, Geneva, Switzerland
RMNH: Nationaal Natuurhistorisch Museum, Leiden, Netherlands
MNHN: Muséum national d’Histoire naturelle, Paris, France
NMPC: National Museum of the Czech Republic, Prague

### 2.3 Molecular analysis

The mesocoxal muscle tissue was excised from specimens preserved in 96% ethanol. Total genomic DNA was extracted using the E.N.Z.A. Tissue DNA Kit protocol for animal tissue. Amplification of a fragment of the cytochrome c oxidase subunit I (*COI*) gene was carried out using the primers LepF1 (5′-ATTCAACCAATCATAAAGATATTGG-3′) and LepR1 (5′-TAAACTTCTGGATGTCCAAAAAATCA-3′) (14) for all specimens processed in the Molecular Laboratory of the SDEI, where the majority of the material was amplified and sequenced by the first author. The specimen from Pakistan, which was available to the fourth author and processed in the Molecular Laboratory of Charles University in Prague, was amplified and sequenced using the primers LCO1490 (5′-GGT CAA CAA ATC ATA AAG ATA TTG G-3′) and HCO2198 (5′-TAA ACT TCA GGG TGA CCA AAA AAT CA-3′) (15). All resulting sequences were of high quality and showed clear chromatograms without ambiguous base calls.

The *COI* barcode region was amplified via polymerase chain reaction (PCR) on a SENSQUEST Lab Cycler. The thermal PCR conditions were as follows: 95°C for 5 min; 38 cycles of 95°C for 30 s, 49°C for 90 s, and 72°C for 60 s; and then 68°C for 30 min. To confirm correct amplification and detect undesired contamination, the PCR products were visualized using gel electrophoresis. Amplicons were purified with the Thermo Fisher Scientific Exonuclease I and FastAP Thermosensitive Alkaline Phosphatase Clean-up Kit. These were sequenced at Macrogen Europe with complements and sufficient overlap with adjacent regions to ensure the accuracy of the sequence data.

The chromatograms were imported into Geneious R10 (https://www.geneious.com) for nucleotide editing, contig assembly, and trimming. Multiple sequence alignment was performed using Bioedit 7.2.5 (16), and the files were converted to Fasta and Nexus formats for use in different analysis programs. A 658-bp *COI* fragment was amplified from 12 individuals of the *Hierodula* species from different Iranian provinces and Pakistan. All sequences were deposited in GenBank (https://www.ncbi.nlm.nih.gov/genbank/) with the following accession numbers: PX649516–PX649527 (**Supplementary File 3**). In addition, five reference sequences were incorporated into the dataset, including two *H. maculata* Wang et al. 2020 specimens (BOLD System: DBMC237-21), two *Hierodula patellifera* Audinet-Serville 1839 specimens (BOLD System: DBMC238-21), and one *Rhombomantis longipennis* (17) specimen (GenBank accession no. OP168283.1), which was used as an outgroup.

For the Bayesian phylogenetic analysis, the best-fit nucleotide substitution model was determined using the modelTest function in the phangorn package in R, which evaluated a range of models (JC, K80, F81, F84, HKY, and GTR, with optional rate heterogeneity and proportion of invariable sites) (18). Model selection was based on the Akaike information criterion (AIC) and Bayesian information criterion (BIC) values. Accordingly, the general time-reversible model with gamma-distributed rate variation among sites (GTR+G) was selected as the best fit to the data (19, 20). This model was then applied to subsequent Bayesian inference analyses using MrBayes 3.2 (21) with default prior settings and partition-specific substitution parameters. All analyses were executed with two independent Markov Chain Monte Carlo (MCMC) runs, each with four chains, sampled every 1,000 generations, for a total of 2 million generations. Convergence was assessed by monitoring the average standard deviation of the split frequencies and by inspecting the parameter trace plots, with the initial 25% of trees discarded as burn-in prior to summary and consensus tree calculation. IQ-TREE 1.6 was used to perform a maximum likelihood (ML) phylogenetic tree estimation (22). In 10 independent runs, 1,000 ultrafast bootstrap (UFBoot) replicates (23) and 1,000 Shimodaira–Hasegawa approximate likelihood ratio test (SH-aLRT) replicates (24) were calculated.

Pairwise genetic distances were computed in R (version 4.2.3) (25) using the ape package (26). The aligned FASTA file was imported as the DNA sequence data, and distance matrices were calculated under multiple nucleotide substitution models commonly applied in molecular systematics. These included the uncorrected *p*-distance model, the Jukes–Cantor model (JC69) (27), the Kimura two-parameter model (K80) (28), and the Tamura–Nei model (TN93) (29). The TN93 model was used as the primary estimator due to its ability to incorporate unequal substitution rates and base frequencies, which provides a more realistic representation of mitochondrial DNA evolution in insects. All distance matrices were exported as comma-separated value (CSV) files for documentation and further analysis. To visualize the divergence patterns among samples, a heatmap of the TN93 distances was generated in R using a custom color gradient. This graphical representation provided an overview of the relative genetic differentiation across specimens of *Hierodula*.

## 3 Results

### 3.1 Analysis of the external morphology

**Figure 1:**
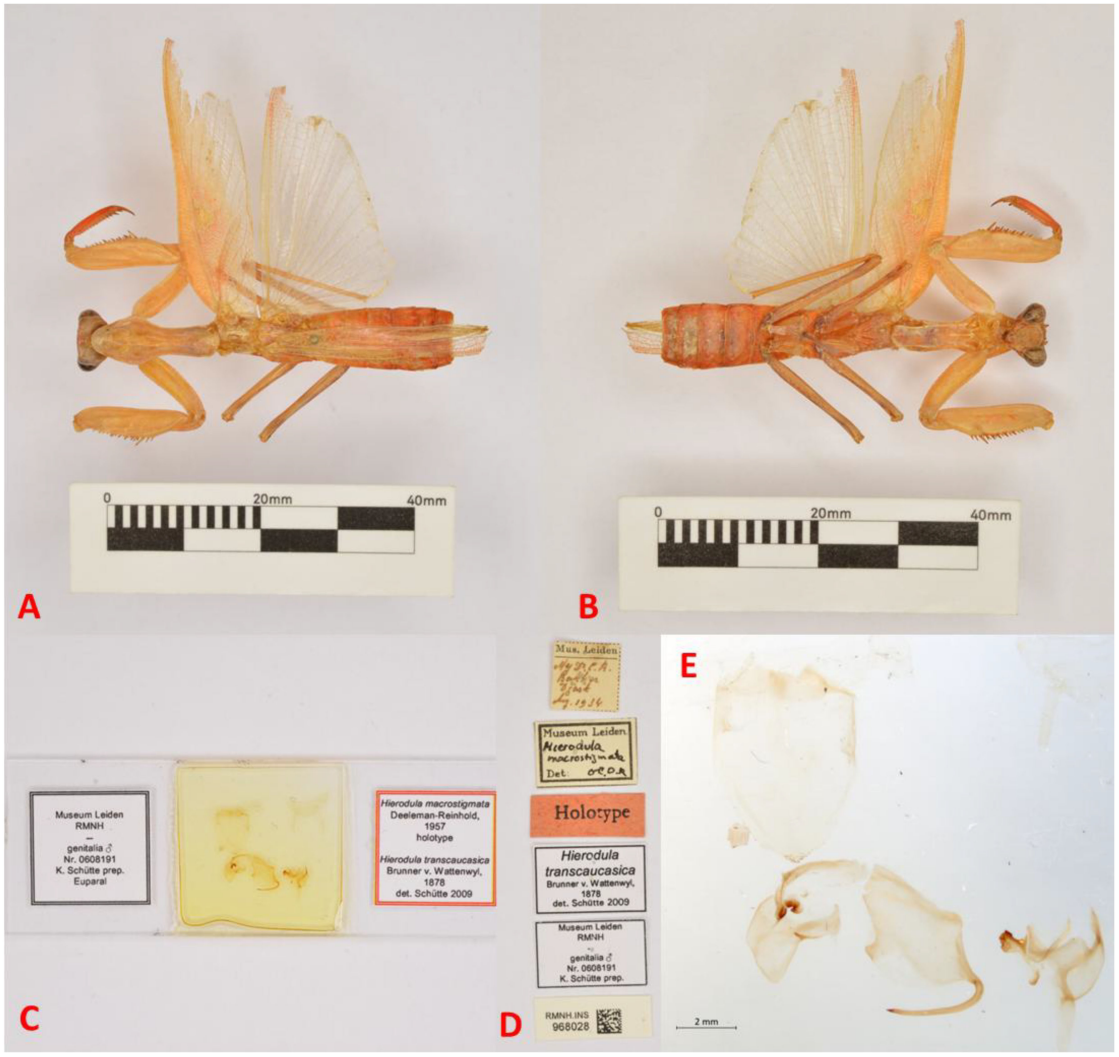
Holotype specimen of *Hierodula macrostigmata*. **(A)** Dorsal aspect. **(B)** Ventral aspect. **(C)** Prepared slide showing the male genitalia. **(D)** Specimen label details. **(E)** Male genitalia observed under a microscope. Photographs by Yvonne van Dam.

**Figure 2:**
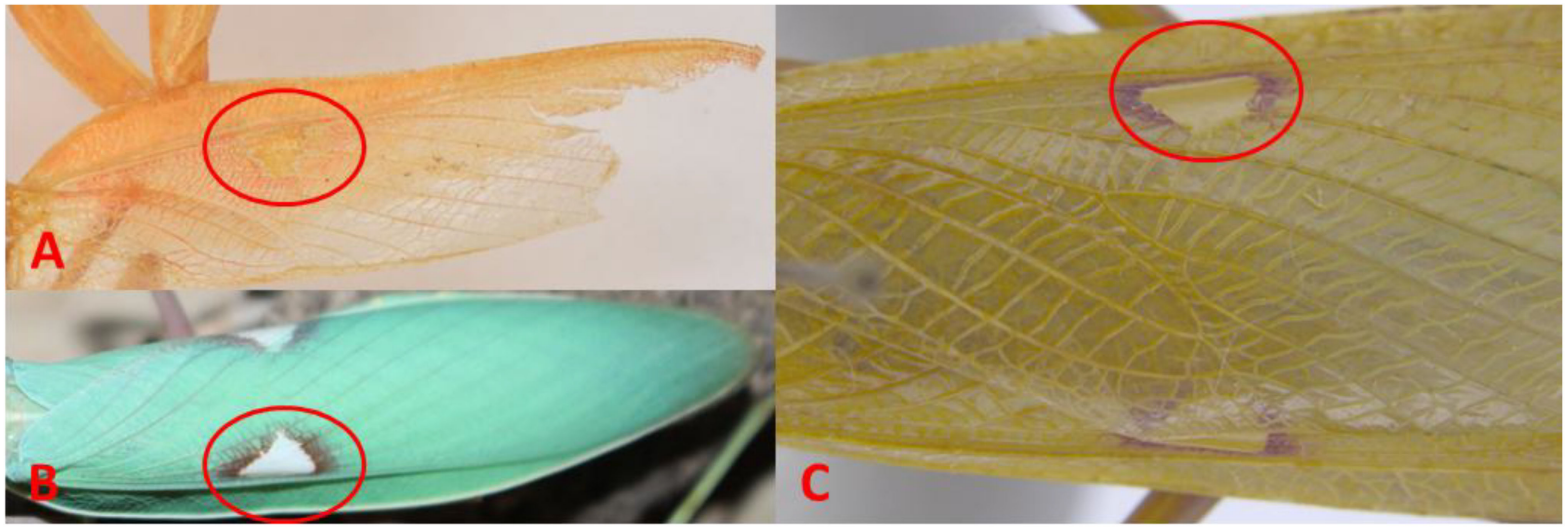
Forewing stigma in *Hierodula coarctata*. **(A)** Holotype of *Hierodula macrostigmata* from Jask, Iran. **(B)** Specimen from Bandar Abbas, Iran. **(C)***H*. *coarctata* from Thambikota, southern India.

**Figure 3:**
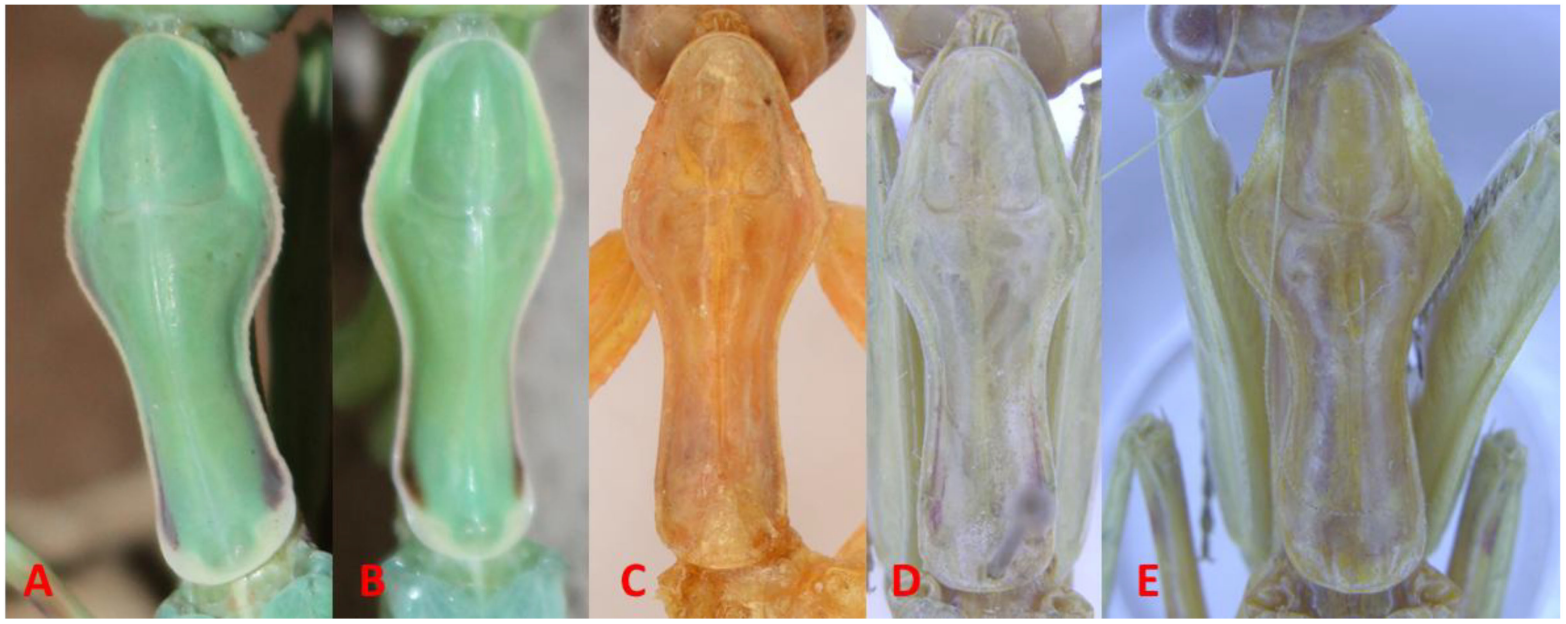
Pronotum shape of *Hierodula coarctata*. **(A)** Female specimen from Bandar Abbas, Iran. **(B)** Male specimen from Bandar Abbas, Iran. **(C)** Holotype of *Hierodula macrostigmata* from Jask, Iran. **(D)** Male specimen from Thambikota, southern India. **(E)** Female specimen from Madras, southern India.

A morphological examination of the holotype of *H. macrostigmata* (**Figure 1**), along with recently collected specimens from Bandar Abbas, Jask, and Iranshahr, and examination of the *Hierodula coarctata* material from India (RHMN) showed a consistent set of morphological traits. All of the examined specimens share a prominent, large, triangular stigma (**Figure 2**) and an elongated, petiolate pronotum with a distinctly narrow anterior “neck” and a broadened, rounded posterior lobe. This spatulate, or paddle-shaped, pronotal form, which is thin at the front and widens toward the base, is characteristic of the genus *Hierodula* and reinforces the morphological match between *H. macrostigmata* and *H. coarctata* (**Figure 3**).

**Figure 4:**
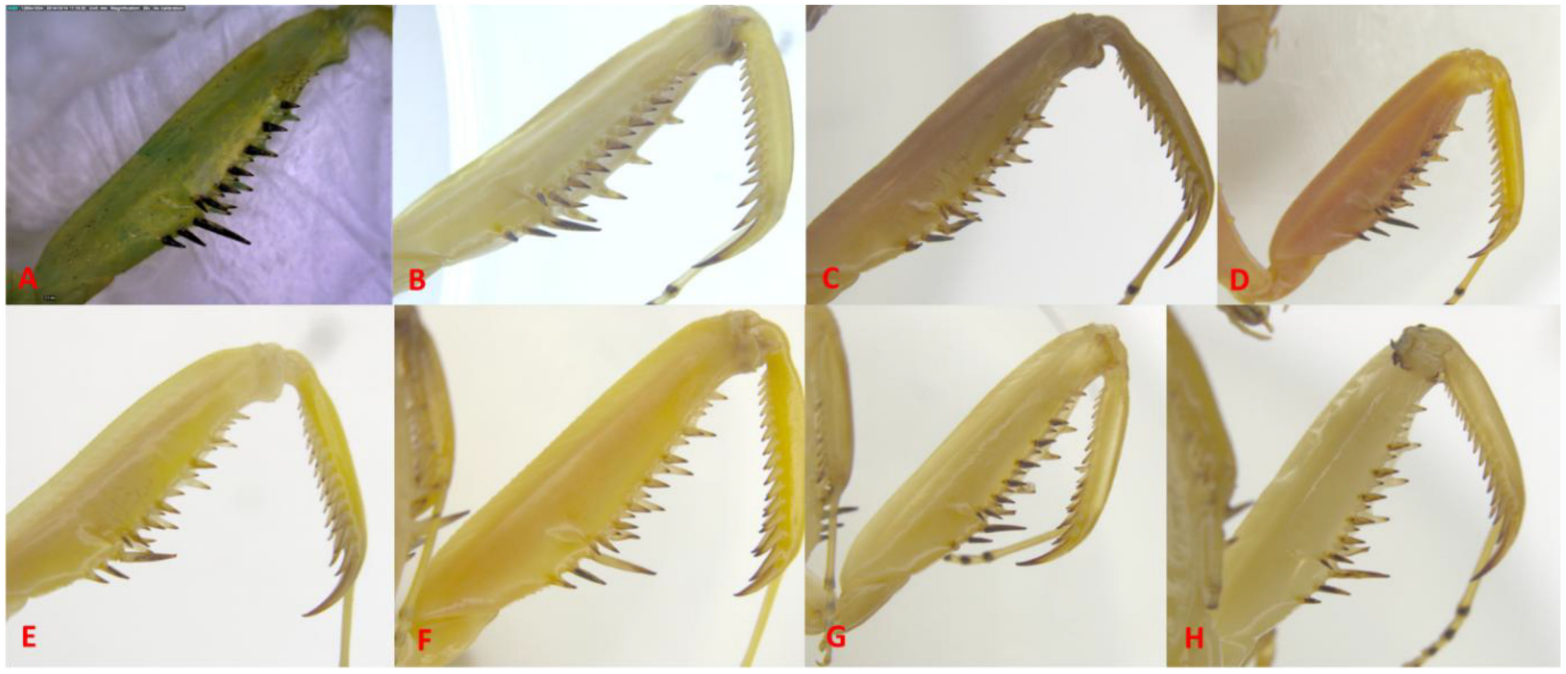
Forefemora of the *Hierodula* specimens examined in this study collected in Tehran Province (Tochal) (ZM-PC777) **(A)**; Gilan Province (Fuman) (ZM-PC538) **(B)**; Fars Province (Kohmare Sorkhi) (ZM-PC544) **(C)**; Golestan Province (Ziyarat Village) (ZM-PC534) **(D)**; Razavi Khorasan Province (Mashhad) (ZM-PC559) **(E)**; Fars Province (Shiraz), (ZM-PC555) **(F)**; Qazvin Province (Qazvin) (ZM-PC541) **(G)**; and Alborz Province (Karaj) (ZM-PC535) **(H)**.

Examination of the *H. transcaucasica* and *H. tenuidentata* specimens revealed that the previously proposed diagnostic features, specifically the key diagnostic feature, i.e., the coloration of the discoidal spines on the fore femora (whether entirely black, as supposedly in *H. transcaucasica*, or black only at the tip, as in *H. tenuidentata*), exhibits broad intraspecific variability among the specimens sampled across all of Iran. **Figure 4** shows some examples of the specimens that were used in this study.

### 3.2 Male genitalia analysis

**Figure 5:**
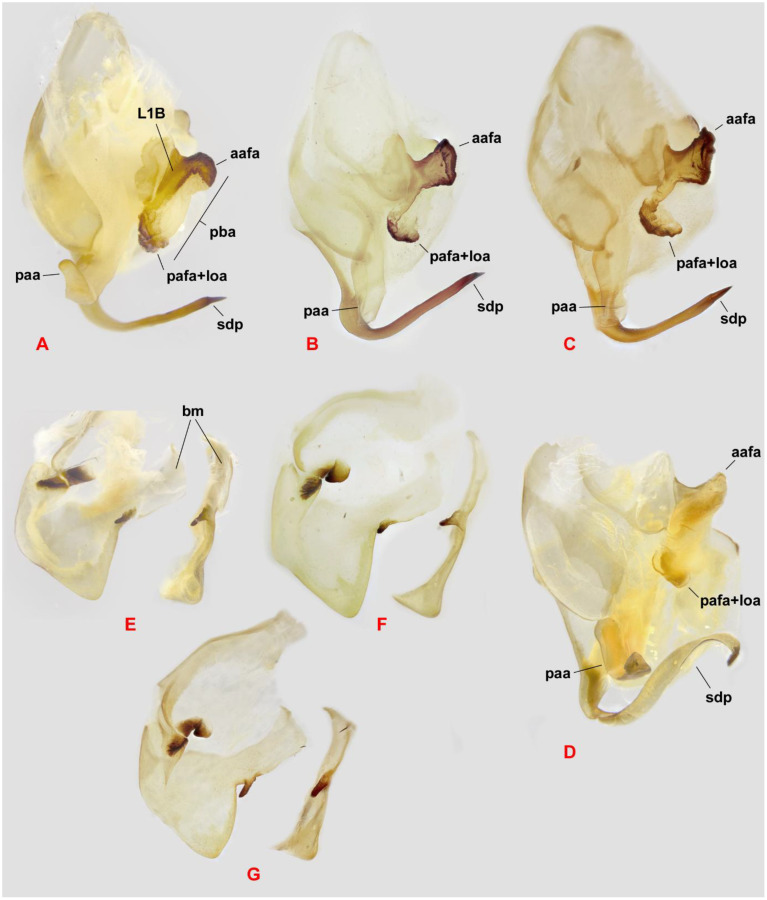
Male genitalia of *Hierodula coarctata*. **(A–D)** Left complex in dorsal view. **(E–G)** Right phallomere in ventral view and edge of the basal arm in left view. **(A, E)** India, Rajasthan (RMNH). **(B, F)** Pakistan (ESPC). **(C, G)** SE, Iran (NMPC). **(D)** Iran, Hormozgan, Bandar Abbas (ZMPC). aafa, paa, pafa+loa, and sdp are processes; bm denotes the basal arm; pba indicates the edge; L1B is the sclerite. Not to scale.

**Figure 6:**
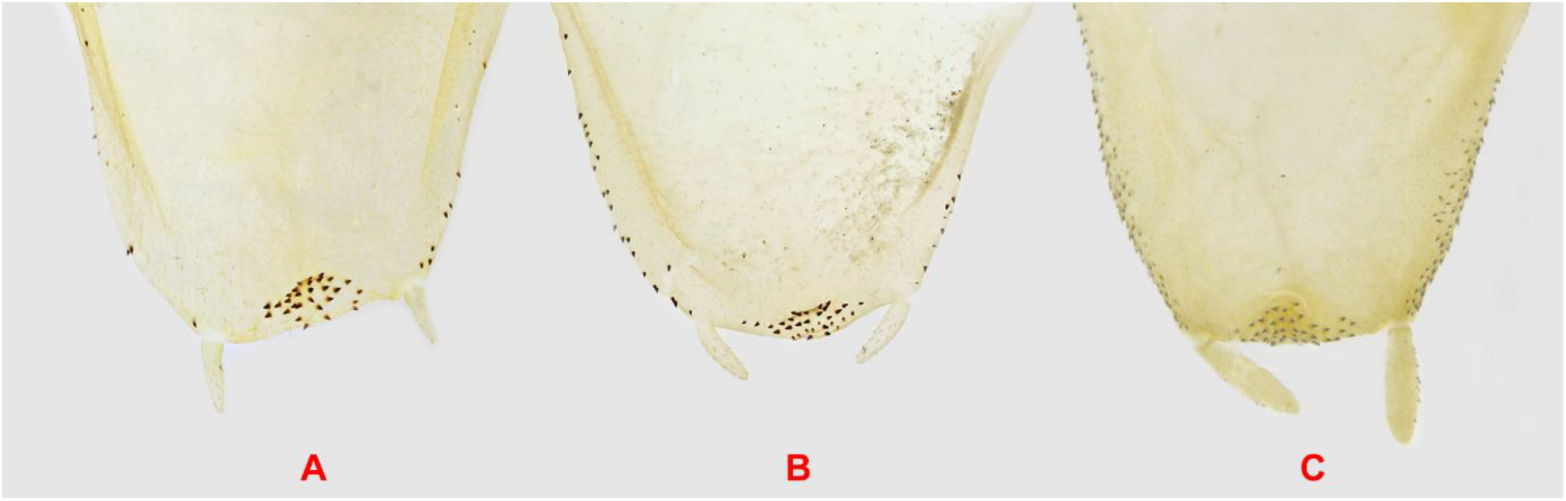
Apex of the male subgenital plate of *Hierodula coarctata* and *Hierodula tenuidentata* in dorsal view. **(A)***H*. *coarctata* from SE Iran (NMPC). **(B)***H*. *coarctata* from Pakistan (ESPC). **(C)***H*. *tenuidentata* from Iran, Golestan, Ziyarat Village (ZMPC). Not to scale.

The male genitalia demonstrate significant variability both within and among populations of *H. coarctata* from various localities (**Figure 5**). In general, the edge pba is very long, with a clearly defined anterior concavity. Process aafa is situated to the right of the concavity, strongly protruding, fully covered by sclerite L1B, widened toward the widely rounded apex, which is covered by small spines, with the dorsal surface of afa mostly smooth. The dorsal «rib» of the edge pba running from aafa to pafa is slightly C-shaped, smooth, and bears a narrow strip of L1B. The process pafa is short, generally rounded at the apex, which is sclerotized by L1B and covered by spines similar to those on the aafa. The paa is moderately wide, with a sharply curved dorsad. The Sdp is moderately thick and typically curved to the right with a straight, with gradually narrowing apex in one specimen from Iran, it is S-shaped (**Figure 5D**). The basal arm of the right phallomere has a finger-like (broad and truncated in one specimen) sclerotized mesal process that is directed posteriad and ventrad and is covered by small spiny tubercles. The subgenital plate (**Figures 6A, B**) is asymmetrical, with the apex inflated and covered by small spines, and the lateral folds are covered by the same spines to varying degrees. The male genitalia of the holotype of *H. macrostigmata* (**Figure 1**) fall well within the normal range of variability for *H. coarctata* specimens.

**Figure 7:**
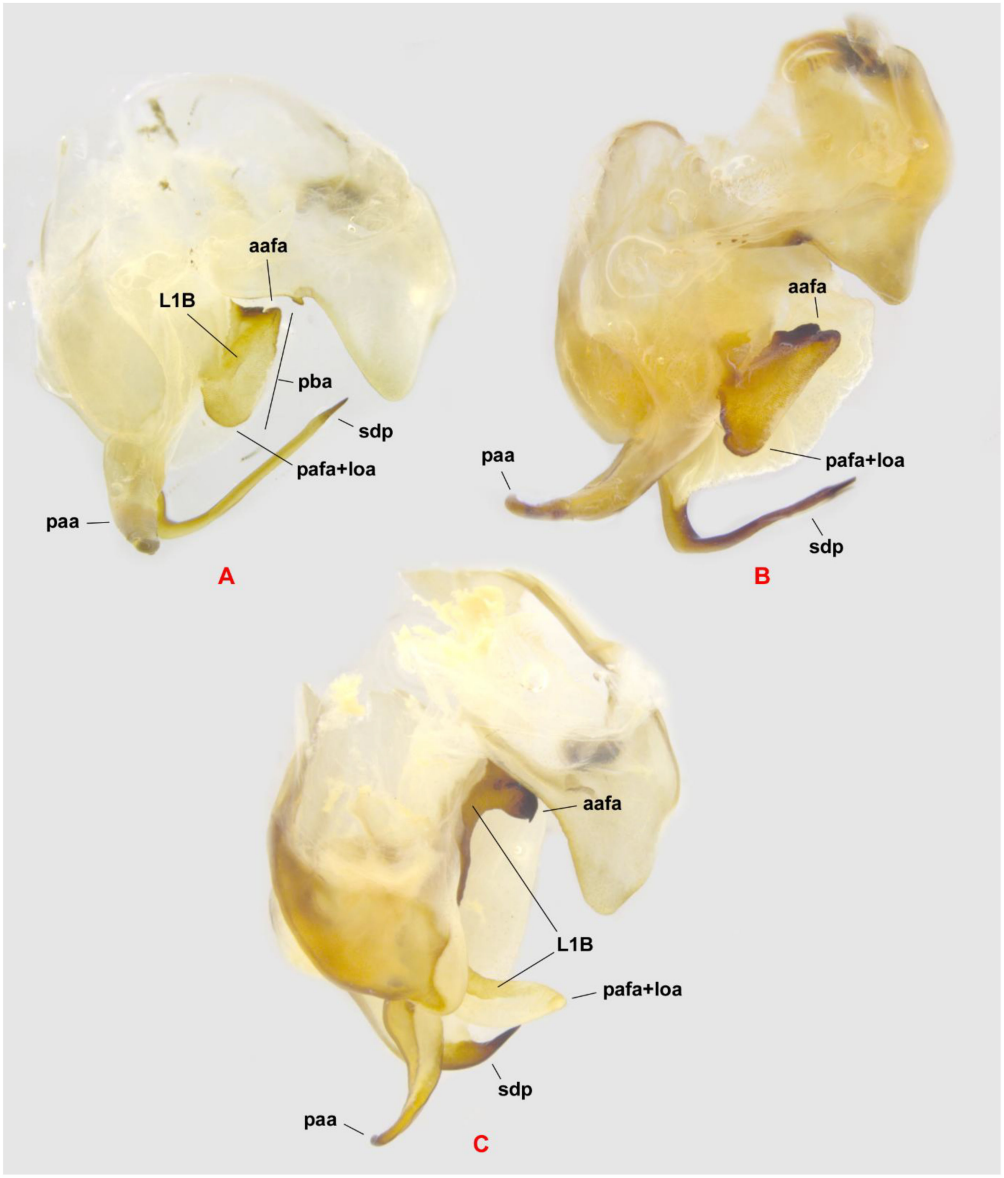
Male genitalia of *Hierodula tenuidentata* and *Sphodromantis trimacula* in dorsal view. **(A)***H*. *tenuidentata* from Iran, Fars, Kohmareh Sorkhi (ZMPC). **(B)***H*. *tenuidentata* from Iran, Golestan, Ziyarat Village (ZMPC). **(C)***S. trimacula* from Oman (ZMPC). aafa, paa, pafa+loa, and sdp denote processes; bm indicates the basal arm; pba is the edge; and L1B is the sclerite. Not to scale.

The diagnostic characteristics of the male genitalia of *H. tenuidentata* are as follows (**Figures 7A, B**). The edge pba is long and straight in-between aafa and pafa, with its anterior part more or less straight and diagonal. The process aafa is short, triangular, and strongly sclerotized by sclerite L1B anteriorly, whereas the right side is less sclerotized; the apex and right side of aafa are covered by small spines. The dorsal surface of afa is mostly smooth. The dorsal «rib» of the edge pba running from aafa to pafa is straight, smooth, and bears a narrow strip of L1B. The process pafa is large and wide, generally reverse bell-shaped, moderately sclerotized by L1B, and covered by spines similar to those on the aafa. The paa is moderately wide, with a curved dorsad. The Sdp is thin, curved to the right with a straight, gradually narrowing apex. The basal arm of the right phallomere, which has a conical, sclerotized mesal process, is directed posteriad and ventrad and is covered by small, spiny tubercles. The subgenital plate (**Figure 6C**) is less asymmetrical, with larger styli and a denser spine cover than that of *H. coarctata*.

### 3.3 Molecular analysis

**Figure 8:**
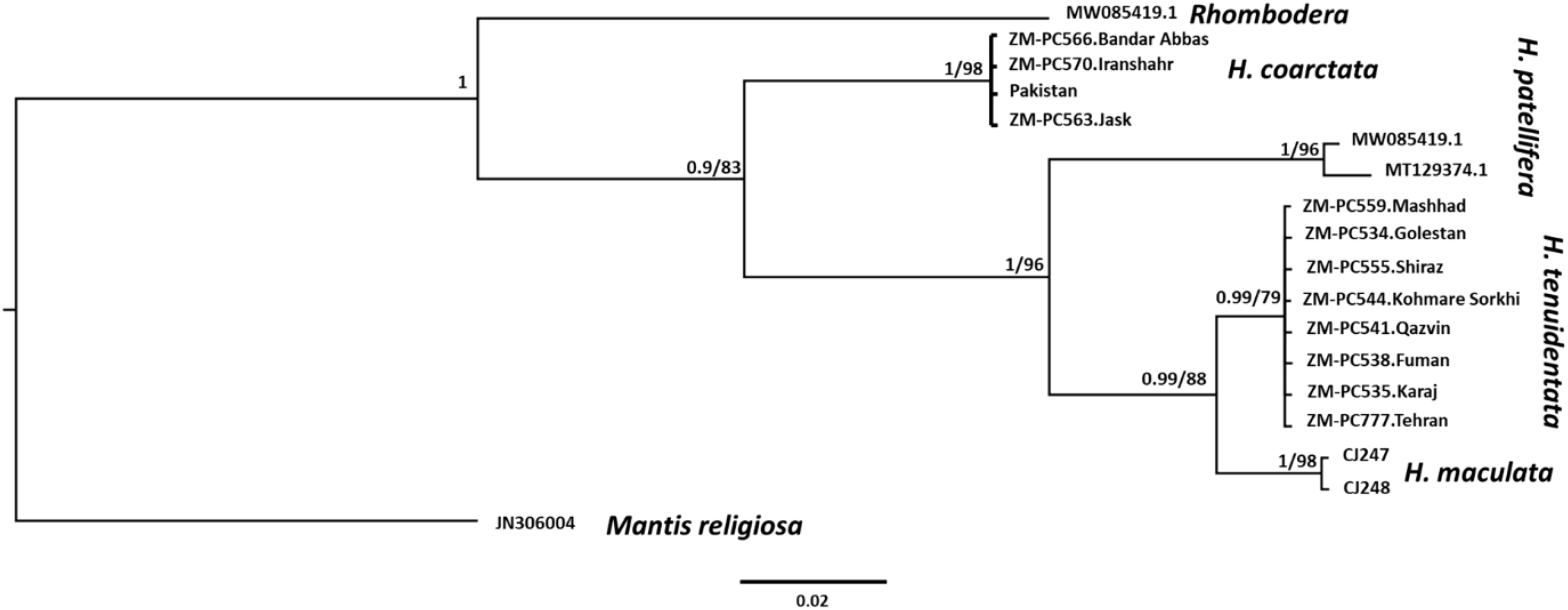
Bayesian inference (BI) and maximum-likelihood (ML) phylogenetic tree of the *Hierodula* species based on the cytochrome c oxidase subunit I (*COI*) sequences. The *node labels* represent the Bayesian posterior probabilities (*left*) and the ML SH-aLRT/ultrafast bootstrap support values (*right*). Specimen codes and GenBank accession numbers are shown for each sample. All species form strongly supported monophyletic clades, consistent with the current taxonomy. The *scale bar* indicates substitutions per site.

Our phylogenetic analysis, based on the *COI* gene, revealed clear and well-supported relationships among the sampled *Hierodula* species and their relatives. The resulting tree (**Figure 8**) was rooted with *Mantis religiosa* (represented by GenBank accession no. JN306004), which formed a distinct outgroup. All *H. coarctata* specimens (ZMPC566, ZMPC570, ESPC0240, and ZMPC563) clustered together in a monophyletic clade (BI PP = 1.0, ML bootstrap = 98). *H. patellifera* (represented by GenBank accession nos. MW085419.1 and MT129374.1) grouped as a highly supported monophyletic lineage (BI PP = 1.0, ML bootstrap = 96), forming a sister group to a clade consisting of *H. maculata* and *H. tenuidentata*. The two *H. maculata* samples (CJ247 and CJ248) were found to be closely related (BI PP = 0.99, ML bootstrap = 88) and divergent from *H. tenuidentata*, which comprised a strongly supported monophyletic group consisting of multiple specimens from all over Iran (BI PP = 0.99, ML bootstrap = 79). The species boundaries inferred from the *COI* data were consistent with the current taxonomy, with all sequences forming discrete, well-supported clades corresponding to the species assignments.

**Figure 9:**
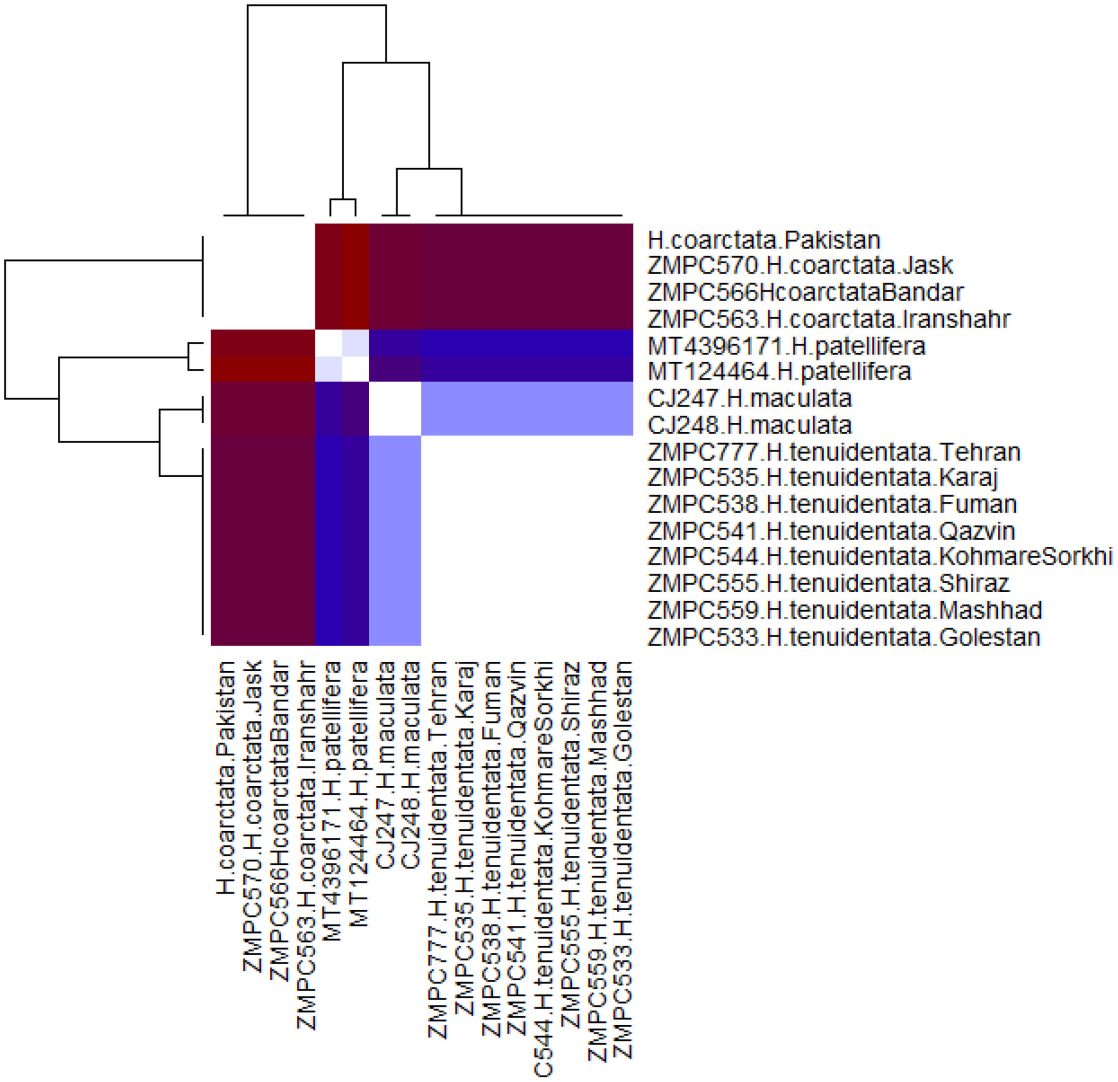
Heatmap showing the pairwise genetic distances calculated using the Tamura–Nei 93 (TN93) model among the *Hierodula* species included in this study. The species are clustered hierarchically, represented by dendrograms. *Colors* indicate the magnitude of genetic distance, ranging from *white* (identical sequences) to *dark red*/*brown* (maximum divergence).

The pairwise genetic distances among the studied *Hierodula* specimens were analyzed using the TN93 model, visualized through a heatmap coupled with hierarchical clustering (**Figure 9**). The color gradient indicates the genetic distance, with white representing identical sequences and colors transitioning from blue to dark red/brown signifying increasing divergence. The analysis clearly resolved the samples into two major, highly divergent clades. The *H. coarctata* clade comprised all individuals identified as *H. coarctata* (ZMPC570, ZMPC566, and ZMPC563) from locations including Pakistan, Jask, Bandar Abbas, and Iranshahr. Members of this clade exhibited low intraspecific distance (light blue/white intersections) but showed a large, significant genetic gap (dark red/blue blocks) when compared against the second major clade. The *H. tenuidentata* complex clade included samples labeled as *H. patellifera*, *H. maculata*, and all *H. tenuidentata* individuals. Within this complex clade, the genetic distances were notably low. Specifically, the two *H. maculata* samples (CJ247 and CJ248) and the eight *H. tenuidentata* samples were genetically similar (light blue and white intersections). Substantial genetic differentiation between the *H. coarctata* clade and the *H. tenuidentata* complex confirms the existence of two distinct molecular operational taxonomic units (MOTUs) among *Hierodula* specimens collected in Iran.

### 3.4 Taxonomy of *Hierodula* in Iran

Order Mantodea Latreille, 1802
Family Mantidae Latreille, 1802
Subfamily Hierodulinae Brunner de Wattenwyl, 1893
Genus *Hierodula* Burmeister, 1838

**Figure 10:**
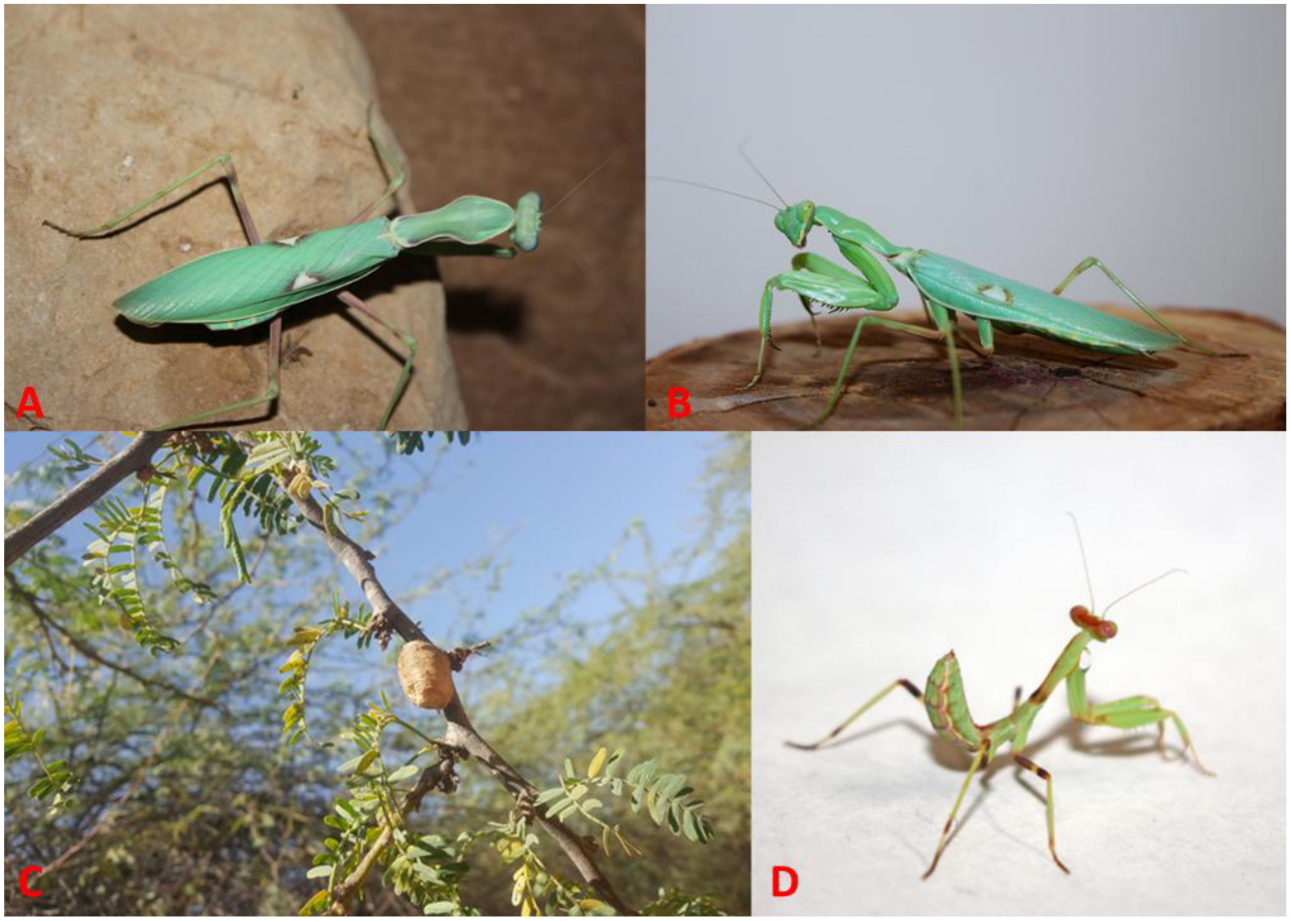
*Hierodula coarctata*. **(A)** Adult female. **(B)** Adult male. **(C)** Oothecae deposited on *Neltuma juliflora*. **(D)** Third-instar nymph. Photographs by Zohreh Mirzaee.

1. *Hierodula coarctata* Saussure, 1869 (**Figures 1**, **5**, **6A, B**, **10**)

*Hierodula coarctata* Saussure. 1869. Mitt. schweiz. ent. Ges. 3:67. Holotype ♂ (MHNG). –India-E

*= Hierodula macrostigmata* Deeleman-Reinhold, 1957 **new. syn.**

*= Hierodula* (*Hierodula*) *macrostigmata* Deeleman-Reinhold, 1957:57–58. Holotype ♂ (RMNH). –Hormozgan Province (Jask), Iran

Material examined. Holotype ♂ of *Hierodula macrostigmata*, Jask, Iran, Aug. 1934 (RMNH) (**Figure 1**); 1♀, Madras, S-India, 1978, leg. Nathan (RMNH); 1♂, Thambikota, S-India, Jul. 1962, leg. Nathan (RMNH); 1♂, Rajasthan; Thar Desert, Barmer, NW-India, Sep. 1973, leg. Nathan (RMNH); 5♀ (ZMPC563, ZMPC564, and ZMPC566–568); 1♂ (ZMPC565), Bandar Abbas, Hormozgan, Iran, 27.187 N, 56.301 E, 9 m, 7. 2022, leg. Mirzaee (ZMPC); 1♂ (ESPC0240), Pakistan, unknown locality and data, local collector (ESPC); 1♂, SE Iran, Tis, 6-7.4.1973 (NMPC).

**Figure 11:**
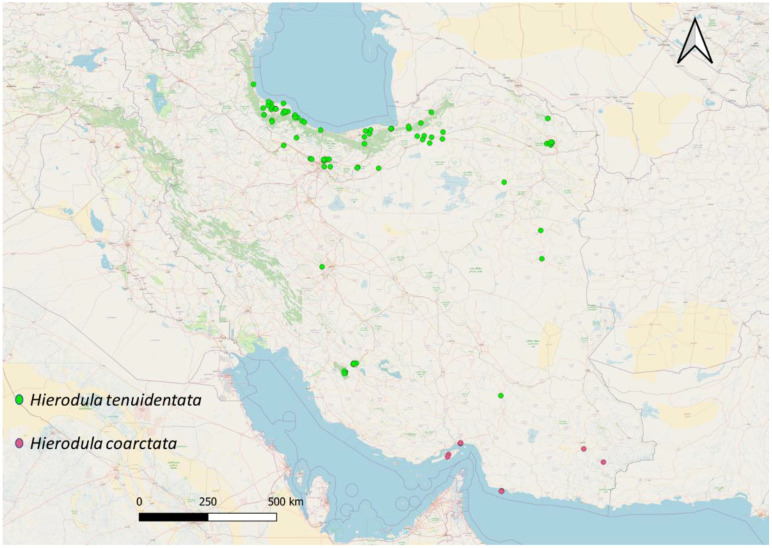
Geographic distribution of the *Hierodula* species in Iran. The sampling localities are indicated by *colored symbols*, representing the different species collected from various provinces.

Distribution in Iran (**Figure 11**). Hormozgan Province [Jask (8), Bandar Abbas, and Qeshm] and Sistan and Baluchestan Province (Iranshahr and Sarbaz). Records from Bandar Abbas, Qeshm, Iranshahr, and Sarbaz are recorded for the first time in Iran by this study.

Global distribution. India (30, 31), Java (32), Nepal (32), Pakistan (33), and Iran (8). Werner (34, 35) reported the species in Indonesia, extremely far from the main distribution. Despite the ease of the species’ identification, these records need to be verified.

Faunal element. Indomalayan

Remarks. A comprehensive examination of newly collected material and a comparison with the holotype of *H. macrostigmata* preserved at the Naturalis Biodiversity Center, Leiden, the Netherlands, along with museum specimens from India and Pakistan, led us to the synonymization of *H. macrostigmata* with *H. coarctata* Saussure, 1869.

**Figure 12:**
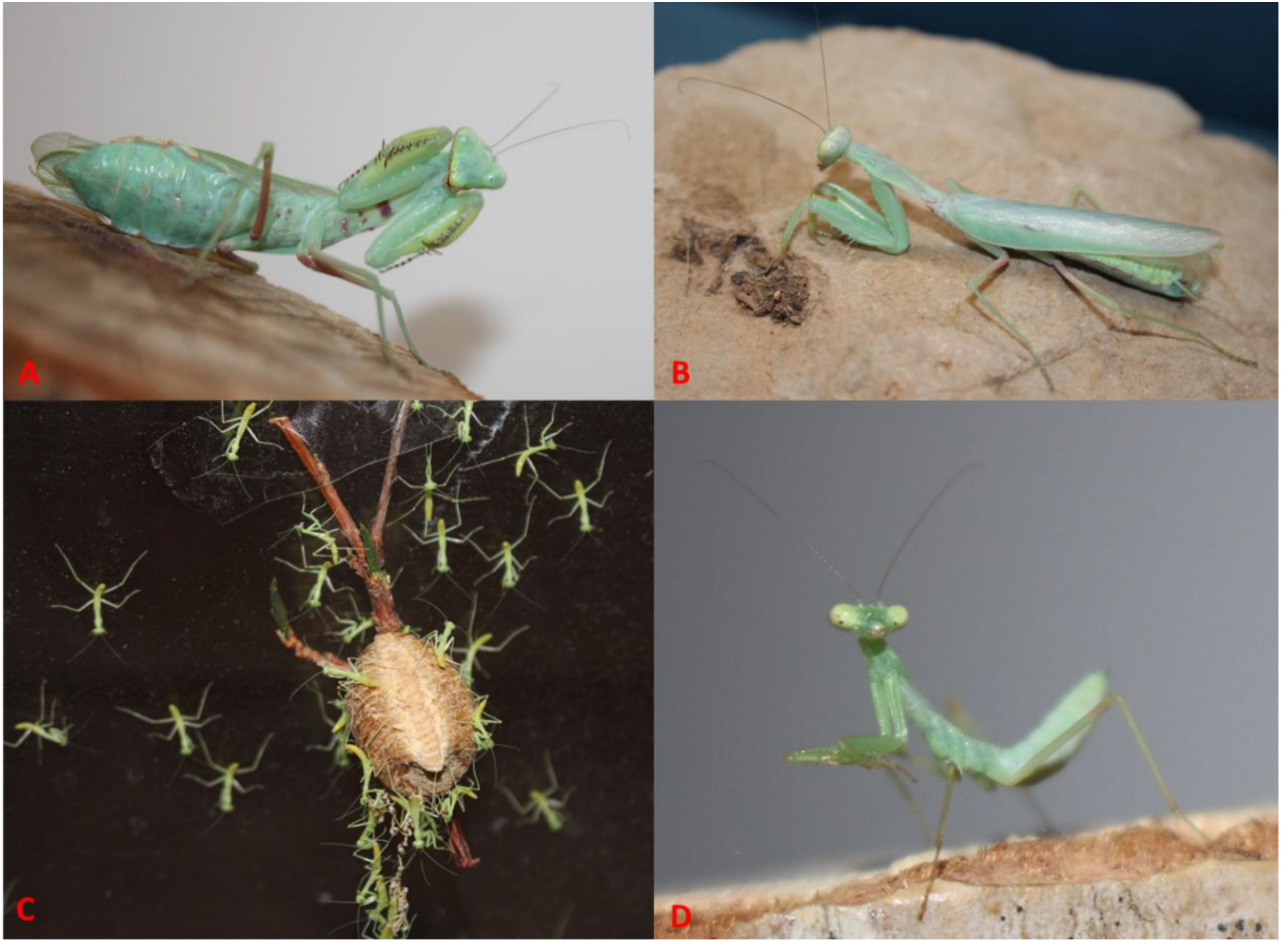
*Hierodula tenuidentata*. **(A)** Adult female. **(B)** Adult male. **(C)** Oothecae with emerged nymphs. **(D)** Second-instar nymph. Photographs by Zohreh Mirzaee.

2. *Hierodula tenuidentata* Saussure, 1869 (**Figures 7A, B**, **6C**, **12**)

*Hierodula tenuidentata* Saussure, 1869: 68. Holotype ♂ (MHNG). –India

= *Sphodromantis* [*Hierodula*] *tenuidentata* (Saussure, 1869): Kirby 1904:244

= *Hierodula westwoodi* Kirby, 1904:247

Material examined. 3♀ (ZMPC576, ZMPC579, and ZMPC580), 2♂ (ZMPC577 and ZMPC578), Tochal, Tehran, Iran, 35.884 N, 51.413 E, 2,701 m, 7. 2016, leg. Mirzaee (ZMPC); 7♂ (ZMPC581, ZMPC585, ZMPC590, ZMPC547, ZMPC548, ZMPC582, and ZMPC583), 5♀ (ZMPC544–546, ZMPC587, and ZMPC588), Kohmareh Sorkhi, Fars, Iran, 29.374 N, 52.162 E, 1,264 m, 8. 2019, leg. Mirzaee (ZMPC); 5♀ (ZMPC552–554, ZMPC591, and ZMPC592), Shiraz, Fars, Iran, 29.699 N, 52.468 E, 1,500 m, 6. 2020, leg. Mirzaee (ZMPC); 1♂ (ZMPC534), Kaboudwall Waterfall, Golestan, Iran, 36.871 N, 54.888 E, 2017, leg. Bakhshi (ZMPC); 1♂ (ZMPC536), Ziyarat Village, Golestan, Iran, 36.705 N, 54.469 E, 2018, leg. Okhli (ZMPC); 2♀ (ZMPC542 and ZMPC543), 4♂ (ZMPC536–539), Fuman, Gilan, Iran, 37.237 N, 49.309 E, 2019, leg. Broomand Fumani (ZMPC); 5♀ (ZMPC555–559), 3♂ (ZMPC560–562), Mashhad, Khorasan Razavi, Iran, 36.297 N, 59.556 E, 2022, leg. Shorabi (ZMPC).

Distribution in Iran (**Figure 11**). North, Northeast, Central, and Southwest Iran.

Global distribution: Borneo, India, Nepal, the Sunda Islands, Tajikistan, Turkmenistan (32), Bosnia and Herzegovina (36), Iran (9, 37), Italy (38), Kazakhstan (39), Romania (40, 41), Serbia (42), Turkey (43), Germany (44), Bulgaria (45), and China (7). Some distribution records, particularly from the Sunda Islands, need verification. See also “*Discussion*.”

Faunal element. Palearctic–Indomalayan.

Subfamily Tenoderinae Brunner de Wattenwyl, 1893

Genus *Sphodromantis* Stål, 1871

**Figure 13:**
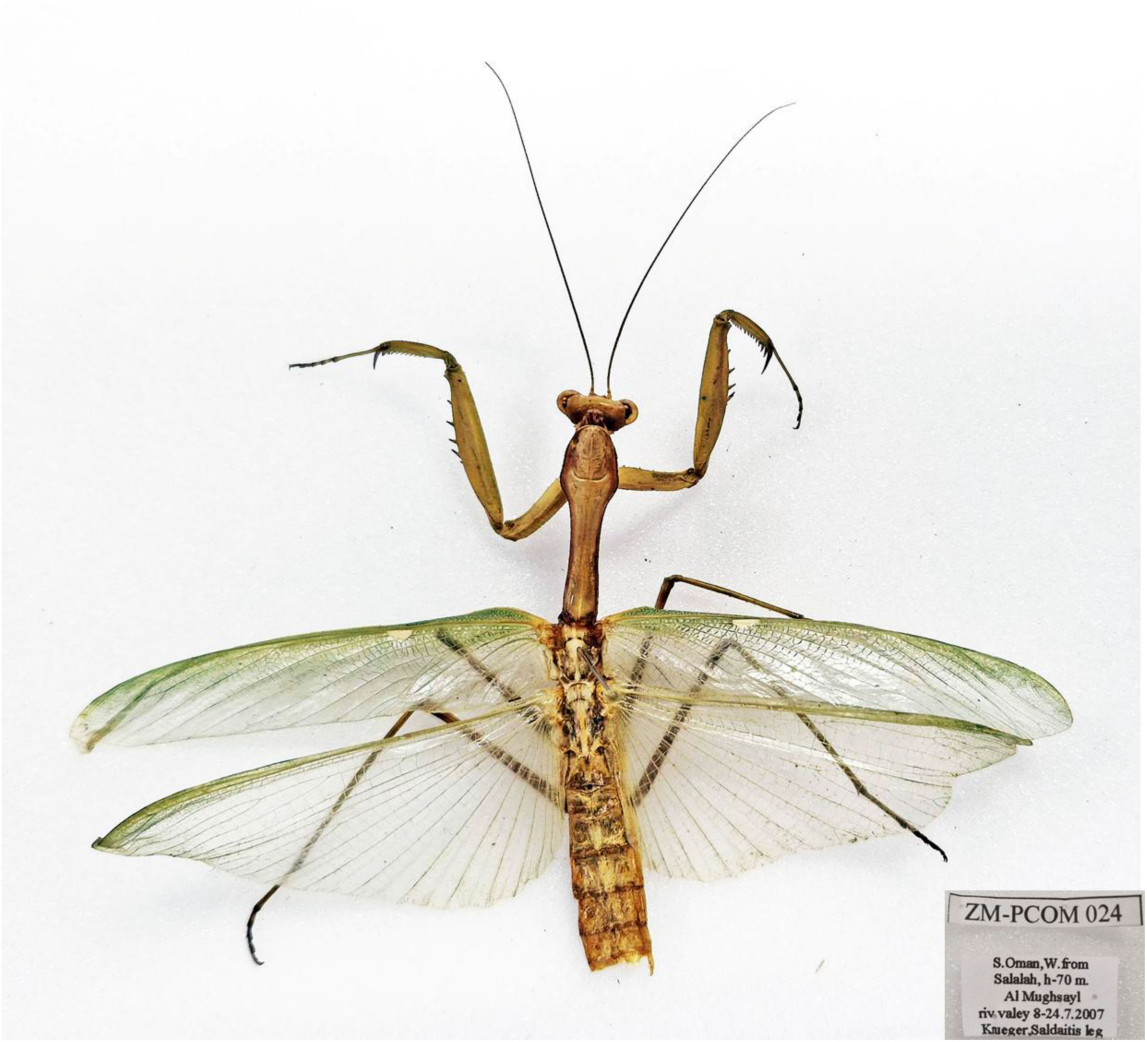
Adult male of *Sphodromantis trimacula* from Oman. Photographs by Zohreh Mirzaee.

*Sphodromantis trimacula* (Saussure, 1870) (**Figures 7C**, **13**)

*Hierodula trimacula* Saussure, 1870: 233. Holotype ♀ (MNHN). –”Sina” (“China”)

= *Hierodula arabica* Wood-Mason, 1882: Uvarov, 1939:554

= *Sphodromantis dhufarica* Uvarov, 1933: Uvarov, 1939:554

= *Sphodromantis trimacula* (Saussure, 1870): Giglio-Tos, 1912:146

Material. 5♂ (ZMPCOM 023–027), Salalah, Oman, 17.019 N, 54.118 E, 70 m, 2007, leg. Krueger Saldaitis (ZMPC); 1♂, Oman, Dhofar Province, Jabal Samhan, 15 km W Jufa, 17.18615° N, 54.94285° E, 380 m, 27.ix.2011, Leg. Walter Grosser (ESPC).

Remarks. The taxonomic placement of *Hierodula*/*Sphodromantis trimacula* has long been a matter of confusion due to historical misidentifications and systematic challenges, as *Sphodromantis* was once considered a subgenus within *Hierodula* (46). Based on our examination of material from Oman, we assigned this species to the genus *Sphodromantis* rather than *Hierodula*. Specifically, the male genitalia possess a flat, lobe-like pafa+loa bearing the stripe of sclerite L1B (**Figure 7C**), characteristic of the genus, as opposed to the broad, bulbous pafa+loa or entirely separate pafa and loa in various species of *Hierodula*. Furthermore, Roy (47) unequivocally identified this species as a member of *Sphodromantis*. Previous literature reflects considerable ambiguity surrounding both its generic placement and its country of origin. Although several previous works, including the Mantodea Species File (1), Giglio-Tos (11), Uvarov (12), Ehrmann (48), and Roy (47), who noted that the specimen label bears the French name “Perse Aucher” (i.e., Persia), have suggested China or Iran as the possible origin of *S. trimacula*, all of these sources ultimately cite only the vague locality “Persia” and provide no precise collection data. Extensive systematic field surveys conducted across Iran over the past 7 years by the first author have not produced any specimens attributable to *S. trimacula*, and no verifiable museum records confirm its presence in Iran. These findings strongly suggest that the historical mention of Persian provenance is a misattribution. Moreover, although “China” is stated in the original description (49) as the origin of the type specimen, newer, reliable collection records of the species are restricted to the Arabian Peninsula, with no members of the genus *Sphodromantis* known from China or adjacent countries. It follows that the type specimen was likely mislabeled, making the actual type locality unidentifiable.

Key to the species of the genus *Hierodula* in the Iranian fauna.

1. Supracoxal dilatation apparent, metazona with constriction. Stigma is very large, triangular, and most often with a brown fringe. Male genitalia with protruding aafa widened toward the apex … *Hierodula coarctata* Saussure, 1869

–. Supracoxal dilatation indistinct, metazona without constriction. Stigma is small, oval, or elongatedly triangular, without a brown fringe. Male genitalia with short, triangular aafa … *Hierodula tenuidentata* Saussure, 1869

### 3.5 Biology and development of the stages of *H. coarctata* from Iran

The field-collected oothecae (**Figure 10C**) were deposited by the females on the branches of the American mesquite [*Neltuma juliflora* (Sw.) Raf.], which is a significant invasive species in southern Iran, particularly in the Hormozgan and Sistan and Baluchestan Provinces. They were found by visually searching the trees. The oothecae are creamy colored and are up to 24 mm in length, of globular shape with a pointed apex. Nymphs hatched from the upper rim of the ootheca. All nymphs from a single ootheca hatched within a single day. Of the 27 collected oothecae, only one was freshly laid and contained unhatched eggs; the other ones were old oothecae from previous years.

A total of 75 nymphs emerged from this single ootheca; of these, 48 nymphs were reared in total. During the first-instar stage, 23 died, and only 25 (two males and 23 females) completed their life cycle in captivity. The nymphs were supplied with an adequate amount of prey (fruit flies and mealworms). There was a high mortality rate from the first to the third instar, which decreased in the last instars. This high mortality rate may have been affected by the diet or other imperfect conditions. The females were more voracious and consumed more prey than the males, and their abdomen was much bigger and broader than that of the males. Males and females of this species are similar in appearance and differ in size and abdomen thickness, with males smaller and more slender than females (**Figure 10**).

## 4 Discussion

This study provides the first integrative revision of the genus *Hierodula* in Iran, combining morphological, molecular, and distributional evidence. Our analyses revealed that only two species, i.e., *H. tenuidentata* and *H. coarctata*, are currently valid for the Iranian fauna. Earlier records of *S. trimacula* from Iran could not be confirmed and are likely the result of historical misidentifications or erroneous attributions.

The synonymization of *H. macrostigmata* with *H. coarctata* is supported by both morphological and molecular evidence. Morphological comparisons between the recently collected Iranian specimens, the holotype of *H. macrostigmata*, and Indian *H. coarctata* material housed in RMNH revealed no distinguishing differences. In addition, *COI* barcode analyses showed that the Iranian material and an *H. coarctata* specimen from Pakistan share identical sequences, forming a single monophyletic clade with no detectable genetic divergence (**Figures 8**, **9**; **Supplementary 2**). This finding not only reduces the number of valid *Hierodula* species in Iran but also extends the known distribution of *H. coarctata* westward into the Iranian Plateau. These results underline the importance of a simple method, such as DNA barcoding, in resolving long-standing taxonomic uncertainties within morphologically conservative mantid groups.

Specimens originally identified as *Hierodula transcaucasica* Brunner von Wattenwyl, 1878, with the type locality of the species in Astrabad (northern Iran), have long posed challenges in terms of their taxonomic identity. The taxonomy of *H. transcaucasica* and *H. tenuidentata* remains uncertain and has been debated for over a decade. Ehrmann (50) originally hypothesized that *H. transcaucasica* might represent a synonym of *H. tenuidentata*, a view later supported by Battiston et al. (38) based on morphological similarities, particularly in the male genitalia, along with variability in forefemoral spine coloration. These findings questioned the validity of traditional diagnostic traits, such as the degree of black pigmentation on discoidal spines, that had long been used to separate the two taxa.

Subsequent literature (e.g., 40, 41, 51) has followed this informal synonymization, while other authors (e.g., 52–54) have preferred to maintain a distinction between the two taxa due to incomplete genetic sampling and the limited morphological coverage across their ranges. A recent niche analysis (55) supported ecological differentiation between Asian and European–Caucasian populations, but did not conclusively resolve their taxonomic relationship.

Our morphological and genetic analyses further support the synonymy between the *Hierodula* specimens collected all over Iran that were previously identified as either *H. transcaucasica* or *H. tenuidentata*. Nevertheless, to determine whether this synonymy should be accepted, we are conducting an ongoing comprehensive taxonomic study involving numerous *Hierodula* specimens from Europe and Asia. This study includes a detailed examination of the genitalia, genetic analyses, and comparative morphology, which may lead to a broader revision of these two *Hierodula* species. Until these analyses are completed, it remains premature to formalize synonymy.

The phylogenetic analysis of *COI* sequences further supports the distinctiveness of *H. tenuidentata* and *H. coarctata*, which form well-supported monophyletic clades. Additional species included for comparative purposes (i.e., *H. patellifera* and *H. maculata*) were clearly differentiated, confirming the reliability of the *COI* data for species delimitation in *Hierodula* species. Nevertheless, deeper relationships among these species were recovered with only moderate support, suggesting that more extensive, multi-locus datasets are necessary to fully resolve the phylogenetic patterns within *Hierodula*.

From a biogeographic perspective, the occurrence of *H. coarctata* in southern Iran is notable. The species was collected in the Hormozgan and Sistan and Baluchestan Provinces, where it appears to exploit the habitats dominated by the invasive tree *N. juliflora*. The association of ootheca deposition with this invasive plant suggests a degree of ecological plasticity that may have facilitated the species’ expansion into new regions. In contrast, *H. tenuidentata* shows a wider distribution across northern, central, and eastern provinces, indicating that it is well adapted to a range of temperate and subtropical habitats in Iran.

In India and Pakistan, both *H. tenuidentata* and *H. coarctata* have been recorded in multiple localities, with checklists and surveys confirming their co-occurrence in several regions and habitats, including agricultural fields and bushy areas (56–59). This sympatry suggests that their ecological requirements and microhabitat preferences are sufficiently broad or differentiated to allow for coexistence, or that competition between these species is minimal. In contrast, the present study’s mapping of Iranian populations reveals that the two species are spatially segregated, with *H. tenuidentata* dominating the northern, central, and eastern regions, while *H. coarctata* is restricted to the southern provinces, such as Hormozgan (**Figure 11**). Environmental or historical factors may have shaped this pattern, possibly related to climatic gradients, habitat structure, or barriers to dispersal within Iran.

This distinctive Iranian pattern raises intriguing questions about the ecological or evolutionary processes underlying range separation. Local adaptations, niche partitioning, or competitive exclusion could be responsible, or the observed pattern may reflect historical colonization routes, barriers (such as mountains or deserts), or recent range shifts. The lack of sympatry in Iran provides an ideal natural setting for niche modeling and further ecological research on the environmental factors that drive the separation of these two species in one part of their range, but the coexistence in another. Understanding these processes may also yield insights into how species interactions and distributions shift under changing climates and landscape connectivity.

Overall, our findings update the taxonomy and distribution of *Hierodula* in Iran. Our study demonstrates the value of combining traditional morphology with molecular tools to disentangle complex species boundaries. Future studies incorporating nuclear markers and broader geographic sampling will be essential to further clarify the evolutionary relationships within *Hierodula* and to understand the ecological factors driving their distribution across the Middle East. Our morphological, ecological, and genetic analyses indicate that all records of the genus *Hierodula* from Iran belong to only two species: *H. tenuidentata* and *H. coarctata*. Taken together, these results support the hypothesis that *H. transcaucasica* is conspecific with *H. tenuidentata* for the Iranian populations thus far. However, given the historical taxonomic complexity of the genus, a comprehensive sampling across its native range (Western, Central, and South Asia) and alien range (South and southern Central Europe) and a detailed integrative revision are required, which will address the formal taxonomic status of these taxa and provide a definitive assessment of their synonymy in an upcoming study.

## Data Availability

The datasets presented in this study can be found in online repositories. The names of the repository/repositories and accession number(s) can be found in the article/**Supplementary Material**.
